# A new method for determination of the photosynthetic pathway in grasses

**DOI:** 10.1007/s11120-019-00646-5

**Published:** 2019-05-15

**Authors:** Marco Antônio Menezes Neto, Miguel Pedro Guerra

**Affiliations:** 1 Faculdade de Ciências Biologia, Instituto de Ciências Biológicas, Universidade Federal do Pará, Belém, Pará Brazil; 2 Laboratório de Fisiologia do desenvolvimento e Genética Vegetal, Centro de Ciências Agrárias, Universidade Federal de Santa Catarina, Florianópolis, Santa Catarina Brazil

**Keywords:** Poaceae, Histochemistry, Safranin, C4, Phenolic, Grasses

## Abstract

An easy and inexpensive method of determining the photosynthetic pathway in grasses using a dye widely used in microscopy. To evaluate the efficiency of a new histochemical test for determination of the photosynthetic pathway in grasses (*Poacea*). Leaves of 58 grass species were sectioned transversally, and the sections treated with a 2% sodium hypochlorite solution to clarify the tissue. After discoloration, sections were washed with distilled water and double-stained with astra blue and safranin (1% each in 50% ethanol) for 1 min. Sections were then mounted between microscopy glass slides and coverslips using water. Grass species showing red staining of the bundle sheath cells were considered C4, and species with translucent bundle sheath were considered C3. The results of the histochemical test were then compared with results from carbon isotope composition analysis and the relevant scientific literature. Observations from the histochemical test were congruent with results from δ^13^C isotope composition analysis, and with data previously presented in the scientific literature. The proposed histochemical test proved efficient for characterization of the photosynthetic pathway in the tested grasses; however, the method should be further tested in a greater number of grass species, encompassing, preferably, all *Poacea* subfamilies. Future studies may elucidate if the proposed method can effectively be used in other botanical families. Furthermore, additional investigations may determine whether the phenolic compounds indicated by the histochemical test are exclusive to the bundle sheath of C4 grasses and if possible relations exist between these phenolic compounds and the C4 photosynthetic pathway in grasses.

## Introduction

The C4 photosynthetic rout evolved in plants tens of millions of years ago in response to environmental changes, characterizing an advantage under high light, hight temperature, or draught conditions (Ehleringer et al. 1991; Bowes 1993; Sage 2004).

There are 19 botanical families with C4 species (Sage et al. 2012). Among them, Poaceae is the most diverse with 4500 C4 species (Christin et al. 2008; Vicentini et al. 2008; Bouchenak-Khelladi et al. 2009; Christin and Besnard 2009), which comprises ca. half of all C4 species (Sage et al. 1999). Within grasses, all C4 lineages occur in branches denominated PACMAD, including the subfamilies Panicoideae, Arundinoideae, Chloridoideae, Micrairoideae, Aristidoideae and Dantoideae (Kellogg 1999, 2001; Sage et al. 2011).

The distinction between C3 and C4 grasses is not often apparent, although some characteristics can aid in the process, with emphasis on: anatomic characters; carbon isotope composition and CO_2_ compensation point; and the activity of specific enzymes. Many authors have attempted to use anatomical characteristics (Hattersley and Watson 1975, 1976; Hattersley and Browning 1981; Hattersley 1984; Prendergast and Hattersley 1987; Prendergast et al. 1987; Dengler et al. 1994), however, evaluation by anatomical traits alone led to much confusion. Carbon isotope composition (δ^13^C) is also used as a distinction criterion between C3 and C4 plants. Values between − 10 and − 14‰ indicate a C4 plant, while values between − 20 and − 35‰ indicate a C3 plant (Cerling 1999); however, this method requires expensive, high-maintenance equipment. Other methods were employed for the determination of photosynthetic pathways in plants (Edwards and Walker 1983; Hatch 1987; Sage et al. 1987), but always with caveats related to time and cost issues.

Neto (2017) proposed a histochemical method for determination of the photosynthetic pathway in grasses using Safranin O. The test is positive for C4 grasses when cells of the bundle sheath stain strongly red. In C3 grasses, cells of the bundle sheath do not stain red. Confirmation of the efficiency of the histochemical test would provide an additional—as well as convenient and cheap method for determination of the photosynthetic pathway in grasses. However, validation of the test requires evaluation of its efficiency in a larger number of grass species, as well as confirmation of whether similar results can be obtained among the three types of C4 grasses [NAD-dependent malic enzyme (NAD-ME), PEP-Carboxikinase (PCK), and NADP-dependent malic enzyme (NADP-ME)], comparing the results from the histochemical test with carbon isotope composition analysis and information within the relevant literature.

## Materials and methods

### Plant material collection and preparation

Control leaf samples were collected from grasses with known photosynthetic pathways (rice—C3; maize and sugarcane—C4 NADP-ME). Leaves of 15 identified forage species and 14 identified bamboo species, of several genera, were collected from plants cultivated at Universidade Federal de Santa Catarina’s Fazenda Experimental da Ressacada, Florianópolis, Santa Catarina, Brazil. Additional leaf samples were obtained from 12 grass species cultivated at Embrapa’s Amazonia Oriental experimental area in Belém, Pará, Brazil, and 13 wild grass species in Santa Catarina state. Dried specimens from grasses collected in Belém were deposited at the Museu Paraense Emílio Goeldi’s herbarium (Herbarium MG), where specimens were identified by means of botanical comparison, and dried specimens of grasses collected in Santa Catarina were deposited at Universidade Federal de Santa Catarina’s herbarium (Herbário Flor), where specimens were also identified by means of comparison.

Leaves collected for determination of carbon isotope composition were placed in paper bags for subsequent dehydration, and leaves used in the histochemical test were fixated in 70% ethanol.

### Determination of stable carbon isotope composition (δ^13^C) in grasses

Leaf samples were dehydrated in a drying stove (50 °C, until constant mass). Afterwards, leaves were ground in liquid nitrogen with a mortar and pestle until a fine powder was obtained. Ground leaf samples were analyzed at the Laboratóro de Ecologia Isotópica—CENA/USP (Piracicaba, SP). About 2.5 mg of leaf material were conditioned inside aluminium capsules and inserted in an element analyser (EA CHN Carlo Erba—modelo E-1110, Milan, Italy), for determination of the total carbon concentration. The gases generated by the samples’ combustion were purified and injected directly into a mass spectrometer (IRMS Delta Plus, Thermo Scientific, Bremen, Germany), for determination of isotope ratios. The natural abundance of ^13^C is expressed as the notation delta (*δ*) and as a value given in parts per thousand (‰), being determined by means of the equation:

$$\delta^{13} {\text{C}} = \frac{{R_{\text{sample}} - R_{\text{standard}} }}{{R_{\text{standard}} }}$$

where *R*_sample_ and *R*_standard_ are the molar ratios ^13^C/^12^C of the sample and the standard, respectively. The employed standard was the fossil limestone PeedeeBelamnite (PDB). At each analysis round reference leaf material of known carbon concentration and δ^13^C was used (sugarcane leaves, δ^13^C = − 12.7 ‰; %C = 43.82%). The acceptable analytical error for carbon concentration determination is 0.3% and for δ^13^C is 0.3‰.

### Histochemical test for determination of the photosynthetic pathway in grasses

Leaves fixed in 70% ethanol were sectioned transversally at their middle portion by hand with stainless steel blades. Leaf sections were treated with 2% sodium hypochlorite solution for tissue clarification. After discoloration, sections were washed with distilled water and double-stained with astra blue and safranin (1% each in 50% ethanol) for 1 min. Sections were then mounted between microscopy glass slides and coverslips, with a drop of water between them. Photomicrographs were obtained using an Olympus BX40 optical microscope attached to an Olympus DP71 camera.

## Results

**Fig. 1:**
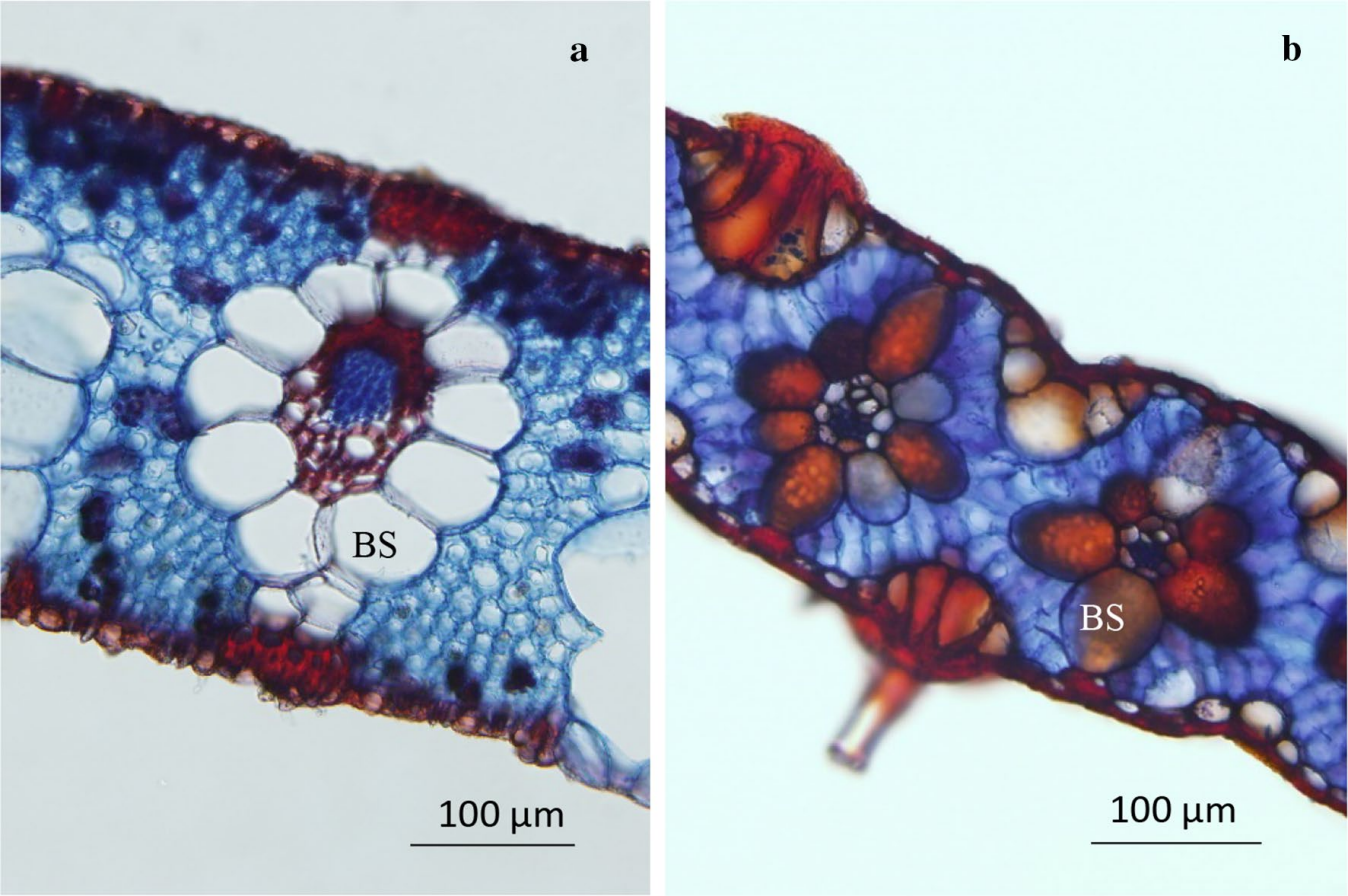
Histochemical test results in *Arundo donax* (**a**) and *Urochloa brizantha* (**b**). The translucent bundle-sheath cells (BS) in *A. donax* and the intensely stained red cells in *U. brizantha* indicate that *A. donax* is C3 and *U. brizantha* is C4

Results from the histochemical test differed between C3 and C4 grasses. C4 grasses presented bundle-sheath cells stained red, while C3 did not. Results for *Arundo donax* and *Uruchloa brizantha* clearly illustrate the distinction (Fig. 1). These results are compatible with those provided in Neto (2017).

Table 1 shows the results of the carbon isotope composition analysis and of the histochemical test of 58 grass species, representing 40 genera, 12 tribes and 6 subfamilies. The results from isotope composition (δ^13^C) analysis of all evaluated grasses correlated with the histochemical test. All grasses with isotope composition values between − 26.09 and − 29.26 indicated a C3 plant, and presented a negative result in the histochemical test. In contrast, all plants with isotope composition values between − 9.88 and − 14.21 indicated a C4 plant, and presented a positive result in the histochemical test.

**Table 1:** Results of the histochemical test (H.T.) and isotopic composition (δ^13^C, average of three replicates and SD) of 58 grass species

Categories/species	Subfamily	Tribe	P.P.	H.T.	δ^13^C‰
Forage					
*Axonopus cothinansis*	Panicoideae	Paniceae	NADP-ME (G)	+	− 12.39 ± 0.29
*Brachiaria brizantha*	Panicoideae	Paniceae	PCK	+	− 11.77 ± 0.17
*Brachiaria decumbens*	Panicoideae	Paniceae	PCK	+	− 12.27 ± 0.27
*Cynodon nlemfluensis*	Chloridoideae	Cynodonteae	NAD-ME (G)	+	− 13.46 ± 0.16
*Festuca arundinacea*	Pooideae	Poeae	C3	−	− 26.09 ± 0.30
*Festuca aurora*	Pooideae	Poeae	C3	−	− 28.14 ± 0.12
*Hemarthria altíssima*	Panicoideae	Andropogoneae	NADP-ME (G)	+	− 11.68 ± 0.24
*Holcus lanatus*	Pooideae	Poeae	C3	−	− 29.26 ± 0.06
*Panicum maximum*	Panicoideae	Paniceae	PCK (E)	+	− 12.45 ± 0.15
*Paspalum atratum*	Panicoideae	Paniceae	NADP-ME (G)	+	− 12.06 ± 0.12
*Paspalum notatum*	Panicoideae	Paniceae	NADP-ME (S)	+	− 11.99 ± 0.13
*Pennisetum americanum*	Panicoideae	Paniceae	NADP-ME (G)	+	− 12.54 ± 0.12
*Pennisetum purpureum*	Panicoideae	Paniceae	NADP-ME (S)	+	− 9.88 ± 0.14
*Pennisetum setaceum*	Panicoideae	Paniceae	NADP-ME (G)	+	n
*Setaria anceps*	Panicoideae	Paniceae	NADP-ME (S)	+	− 11.04 ± 0.12
*Sorghum sudanense*	Panicoideae	Andropogoneae	NADP-ME (G)	+	− 12.80 ± 0.09
Bamboo					
*Bambusa oldhamii*	Bambusoideae	Bambuseae	C3	−	− 28.66 ± 0.23
*Chimono bambusa quadrangulares*	Bambusoideae	Bambuseae	C3	−	− 31.01 ± 0.10
*Chusquea leptophyla*	Bambusoideae	Bambuseae	C3	−	− 27.92 ± 0.18
*Dendrocalamus asper*	Bambusoideae	Bambuseae	C3	−	− 26.42 ± 0.12
*Drepanostachyum falcatum*	Bambusoideae	Arundinarieae	C3	−	− 29.12 ± 0.12
*Fargesia gaolinensis*	Bambusoideae	Arundinarieae	C3	−	− 27.71 ± 0.07
*Guadua chacoensis*	Bambusoideae	Bambuseae	C3	−	− 30.83 ± 0.19
*Parodiolyra micrantha*	Bambusoideae	Olyraceae	C3	−	− 28.70 ± 0.20
*Phyllostachys nigra nigra*	Bambusoideae	Bambuseae	C3	−	− 29.36 ± 0.16
*Pleioblastus simonii*	Bambusoideae	Bambuseae	C3	−	− 28.05 ± 0.15
*Pseudosasa japônica*	Bambusoideae	Bambuseae	C3	−	− 29.15 ± 0.15
*Raddia soderstromii*	Bambusoideae	Olyreae	C3	−	− 29.16 ± 0.08
*Sasa palmate*	Bambusoideae	Bambuseae	C3	−	− 29.91 ± 0.18
* Thyrsostachys siamensis*	Bambusoideae	Bambuseae	C3	−	− 28.99 ± 0.09
Collected					
*Arundo donax*	Arundinoideae	Arundineae	C3	−	− 30.30 ± 0.09
*Cenchrus echinatus*	Panicoideae	Paniceae	NADP-ME (G)	+	− 12.12 ± 0.14
*Cymbopogon citratus*	Panicoideae	Andropogoneae	NADP-ME (G)	+	− 12.4 ± 0.20
*Digitaria ciliares*	Panicoideae	Paniceae	NADP-ME (G)	+	− 11.99 ± 0.18
*Digitaria horizontalis*	Panicoideae	Paniceae	NADP-ME (G)	+	− 12 ± 0.20
*Echinochloa colona*	Panicoideae	Paniceae	NADP-ME (G)	+	− 13.02 ± 0.14
*Echinochloa crus*-*pavonis*	Panicoideae	Paniceae	NADP-ME (G)	+	− 12.64 ± 0.16
*Echinochloa polystachya*	Panicoideae	Paniceae	NADP-ME (G)	+	− 11.88 ± 0.26
*Eleusine indica*	Chloridoideae	Cynodonteae	NAD-ME (G)	+	− 13.3 ± 0.09
*Eragrostis tenufolia*	Chloridoideae	Eragrostideae	NAD-ME (G)	+	− 14.21 ± 0.07
*Homolepis aturensis*	Panicoideae	Paniceae	C3	−	− 28.8 ± 0.12
*Homolepis isocalycia*	Panicoideae	Paniceae	C3	−	− 26.3 ± 0.13
*Hymenachne amplexicaulis*	Panicoideae	Paniceae	C4	+	− 12 ± 0.14
*Megathyrsus maximus*	Panicoideae	Paniceae	C4 (G)	+	− 13.47 ± 0.24
*Paspalum conjugatum*	Panicoideae	Paniceae	NADP-ME (G)	+	− 12.3 ± 0.13
*Paspalum maritimum*	Panicoideae	Paniceae	NADP-ME (G)	+	− 11.6 ± 0.18
*Paspalum melanospermum*	Panicoideae	Paniceae	NADP-ME (G)	+	− 11.8 ± 0.09
*Paspalum pauciciliatum*	Panicoideae	Paniceae	NADP-ME (G)	+	− 13.54 ± 0.12
*Paspalum urvillei*	Panicoideae	Paniceae	NADP-ME (G)	+	− 12.37 ± 0.09
*Paspalum virgatum*	Panicoideae	Paniceae	NADP-ME (G)	+	− 11.9 ± 0.13
*Setaria parviflora*	Panicoideae	Paniceae	NADP-ME (G)	+	− 12.19 ± 0.18
*Sorghum halepense*	Panicoideae	Andropogoneae	NADP-ME (G)	+	− 13.11 ± 0.13
*Sporobolus indicus*	Chloridoideae	Zoysieae	NAD-ME (G)	+	− 13.3 ± 0.21
*Steinchisma laxum*	Panicoideae	Paniceae	C3	−	− 27 ± 0.21
*Urochloa brizantha*	Panicoideae	Paniceae	PCK (G)	+	− 13.02 ± 0.18
Crops					
*Oryza sativa*	Oryzoideae	Oryzeae	C3	−	n
*Saccharum officinarum*	Panicoideae	Andropogoneae	NADP-ME (G)	+	n
*Zea mays*	Panicoideae	Andropogoneae	NADP-ME (S)	+	n

It includes subfamily, tribe, and the photosynthetic pathway (P.P., C3 or C4) and the C4 type of some species (S) and genera (G), according to the literature data (Sage et al. 1999)

*n* not determined

The histochemical test showed a negative result for all evaluated bamboos, and confirmated the photosynthetic pathway of maize and sugarcane (C4 type NADP-ME, with positive results) and rice (C3, with a negative result), and positive to the three photosynthetic pathway grasses (PDK, NAD-ME and NADP-ME) (Table 1).

Among the forage grasses, only *Festuca aurora*, *Festuca arundinacea*, and *Holcus lanatus* showed negative results. Among the tested wild grasses, *Homolepsis aturensis*, *H. isocalycia*, *Steinchis malaxum*, and *A. donax* showed negative results, indicating a C3 photosynthetic pathway. All other tested species of forage and wild grasses presented a positive result, indicating they have C4 photosynthetic systems.

Leaf transversal sections that underwent dehydration during performance of the histochemical test presented a slight detachment of the cell wall membrane, and this allowed inferring that the safranin-reactive phenolic substance is located in C4 grasses’ bundle sheath cells’ protoplasts, and not in the cell wall (data not shown).

## Discussion

Safranin O is the least specific histochemical indicator of lignin because it not only stains lignin, but all related phenolic substances (Lewis and Yamamoto 1990). The red staining exclusively observed in cells from the bundle sheath of C4 grasses indicates the existence of a phenolic substance only in these cells.

Many plant species present hydroxycinnamic acids (ρ-coumaric, sinapic and ferulic acids) attached to cell walls (Chen et al. 1998; Brett et al. 1999; Bunzel et al. 2003). Derived from the phenilpropanoid metabolism (Ou and Kwok 2004), ferulic acid (4-hydroxy-3-methoxycinnamic) is abundant in plant epidermis, xylem, sheath bundle, and sclerenchyma cells (Faulds and Williamson 1999; Lambert et al. 1999), while also being present on primary and secondary cell walls in grasses (Harris and Hartley 1976).

The red stain observed in bundle sheath cells may indicate the presence of hydroxicinamic acid. However, the observation that the phenolic substances revealed by the histochemical test are located in the protoplasts of bundle sheath cells, and not in the cell wall, suggests such substances are likely to perform another important function inside the cells. The presence of these substances exclusively in cells of C4 grasses’ bundle sheaths may indicate an antioxidant function in view of the capacity antioxidant of these substance. According to Faulds and Williamson (1999) hydroxycinnamic acids, such as ferulic, sinapic, cafeic and p-coumaric acid, are found both covalently attached to the plant cell wall and as soluble forms in the cytoplasm.

The results of the histochemical test of bamboos were expected, given that bamboos are included in the Bambusoideae subfamily, which is composed exclusively of C3 species, distributed between the Bambuseae, Olyreae e Arundinarieae tribes.

The genera *Festuca* and *Holcus* are included in the Pooideae subfamily, which is composed exclusively by C3 species (Sage and Monson 1999). *Homolepis aturensis*, *Homolepis isocalycia* e *Steinchis malaxum* are part of the Panicoideae subfamily, Paniceae tribe. The Paniceae tribe presents C3, C4 (of all biochemical subtypes) and intermediary C3–C4 plants. *Arundo donax* is included in the Arundinoideae subfamily (composed of 60 genera, of which five are C4) and within the Arundineae tribe, composed exclusively of C3 species (Sage et al. 1999).

The genera *Axonopus, Cenchrus, Cymbopogon, Digitaria, Hemarthria, Paspalum, Pennisetum, Setaria* and *Sorghum* are C4 of the NADP-ME type. The photosynthetic pathway NADP-ME occurs in the forage species *Setaria anceps, Pennisetum purpureum*, and *Paspalum notatum*. The genera *Brachiaria* and *Urochloa* are C4 of type PCK, and genera *Cynodon*, *Eragrostis* and *Sporobolus* are C4 of type NAD-ME. The genus *Panicum* is the only genus to present C3, C4 of all types, and intermediary C3-C4 species, with *Panicum maximum* being PCK (Sage et al. 1999).

The results from the histochemical test and the carbon isotope composition analysis are in accordance with previous reports concerning the plant genera represented in this study, indicating that the proposed histochemical test can be employed as an additional criterion for the determination of the photosynthetic pathway in grasses. The convenience and low cost of the proposed method allows its utilization as a first indicator of the photosynthetic pathway in grasses. However, its use is recommended only with fresh samples, given that its performance with dry samples is not feasible.

Future studies may confirm the applicability of this method in other botanical families with C4 member species. In Cyperaceae, preliminary studies did not validate use of the test (data not shown). Additionally, the isolation and identification of the phenolic compounds detected by the histochemical test in bundle sheath cells of C4 grasses may elucidate a possible relation between the presence of these substances and the C4 photosynthetic pathway.
